# The Impact of Matrix Metalloproteinase-11 Polymorphisms on Colorectal Cancer Progression and Clinicopathological Characteristics

**DOI:** 10.3390/diagnostics12071685

**Published:** 2022-07-11

**Authors:** Hsien-Cheng Huang, Bei-Hao Shiu, Shih-Chi Su, Chi-Chou Huang, Wen-Chien Ting, Lun-Ching Chang, Shun-Fa Yang, Ying-Erh Chou

**Affiliations:** 1 Institute of Medicine, Chung Shan Medical University, Taichung 402, Taiwan; asiantcumed@gmail.com (H.-C.H.); shiubeihao@gmail.com (B.-H.S.); 2 Department of Emergency Medicine, Kuang Tien General Hospital, Taichung 433, Taiwan; 3 Department of Surgery, Chung Shan Medical University Hospital, Taichung 402, Taiwan; hcjy341@gmail.com (C.-C.H.); tingwenchien@gmail.com (W.-C.T.); 4 School of Medicine, Chung Shan Medical University, Taichung 402, Taiwan; 5 Whole-Genome Research Core Laboratory of Human Diseases, Chang Gung Memorial Hospital, Keelung 204, Taiwan; ssu1@cgmh.org.tw; 6 Department of Dermatology, Drug Hypersensitivity Clinical and Research Center, Chang Gung Memorial Hospital, Linkou 333, Taiwan; 7 Department of Mathematical Sciences, Florida Atlantic University, Boca Raton, FL 33431, USA; changl@fau.edu; 8 Department of Medical Research, Chung Shan Medical University Hospital, Taichung 402, Taiwan

**Keywords:** colorectal cancer, MMP-11, polymorphism

## Abstract

Colorectal cancer (CRC) is the third most common cause of cancer mortality worldwide and the most prevalent cancer in Taiwan. The matrix metalloproteinase (MMP)-11 is a proteolytic enzyme of the MMP family which is involved in extracellular matrix degradation and tissue remodeling. In this study, we focused on the associations of *MMP-11* single-nucleotide polymorphisms (SNPs) with CRC susceptibility and clinicopathological characteristics. The *MMP-11* SNPs rs131451, rs738791, rs2267029, rs738792, and rs28382575 in 479 controls and 479 patients with CRC were analyzed with real-time polymerase chain reaction. We found that the *MMP-11* SNP rs738792 “TC + CC” genotype was significantly associated with perineural invasion in colon cancer patients after controlling for clinical parameters [OR (95% CI) = 1.783 (1.074–2.960); *p* = 0.025]. The *MMP-11* rs131451 “TC + CC” genotypic variants were correlated with greater tumor T status [OR (95% CI):1.254 (1.025–1.534); *p* = 0.028] and perineural invasion [OR (95% CI):1.773 (1.027–3.062); *p* = 0.040) in male CRC patients. Furthermore, analyses of The Cancer Genome Atlas (TCGA) revealed that MMP-11 levels were upregulated in colorectal carcinoma tissue compared with normal tissues and were correlated with advanced stage, larger tumor sizes, and lymph node metastasis. Moreover, the data from the Genotype-Tissue Expression (GTEx) database exhibited that the *MMP-11* rs738792 “CC” and “CT” genotypic variants have higher MMP-11 expression than the “TT” genotype. In conclusion, our results have demonstrated that the *MMP-11* SNPs rs738792 and rs131451 may have potential to provide biomarkers to evaluate CRC disease progression, and the *MMP-11* rs131451 polymorphism may shed light on sex discrepancy in CRC development and prognosis.

## 1. Introduction

Colorectal cancer (CRC), which is the third leading cause of cancer death worldwide, ranks as the third most common adult cancer in men the second most common in women [1,2,3,4]. In Taiwan, the incidence of CRC is the highest among all cancers, and CRC is responsible for the third highest cancer mortality [5]. Risk factors such as age, male sex, family history of colorectal cancer, race and ethnicity, cigarette smoking, excessive alcohol consumption, high consumption of red and processed meat, diabetes, obesity, and inflammatory bowel disease were suggested to be associated with CRC carcinogenesis [6,7,8,9,10,11,12,13,14,15,16,17].

The matrix metalloproteinases (MMPs) are a zinc-dependent endopeptidases family which is involved in extracellular matrix (ECM) degradation and tissue remodeling [18,19,20,21]. MMP-11, or stromelysin 3, is a proteolytic enzyme which belongs to the MMP family [22,23,24]. Previous studies have suggested that the MMP-11 may play a regulatory role in cancer cell growth, tumor migration, invasion, and metastasis in various cancers [22,24,25,26,27,28]. In colorectal cancer, it was suggested that MMP-11 is highly expressed in colonic carcinoma [29], and the elevated serum levels and mRNA expression of MMP-11 were correlated with poor prognosis in colon cancer patients [30,31]. Single-nucleotide polymorphisms (SNPs) are the most common type of genetic variations which may lead to amino acid substitution and alteration of protein function [32,33]. The *MMP-11* SNPs were found to be associated with increased risk of cancer progression and development, metastasis, recurrence, or poor prognosis in many cancers including oral squamous cell carcinoma (OSCC) [34], hepatocellular carcinoma (HCC) [35,36], prostate cancer [37], and urothelial cell carcinoma (UCC) [38]. However, the associations and influences of *MMP-11* polymorphisms regarding CRC tumor progression and clinicopathologic characteristics remained uninvestigated. In this study, we focused on five SNPs of *MMP-11* rs131451, rs738791, rs2267029, rs738792, and rs28382575, and try to unveil their correlations to CRC susceptibility and clinicopathologic characteristics.

## 2. Materials and Methods

### 2.1. Subjects

In this study, we enrolled 479 CRC patients and 479 cancer-free controls. All participants were recruited from 2016 to 2020 at Chung Shan Medical University Hospital in Taichung, Taiwan. According to the American Joint Committee on Cancer (AJCC) [39], the TNM staging of the CRC patients who enrolled in our study were staged clinically at the time of diagnosis. The tumor differentiation was examined and rated under the AJCC classification by a pathologist. The demographic data of age and gender were reported by each participant and recorded. Individuals with neither a history of cancer of any sites nor any self-reported diseases, such as asthma, diabetes, or cardiovascular, autoimmune, and neurological diseases, were enrolled in the control group. This project was approved by the institutional review board of Chung Shan Medical University Hospital (IRB number CS1-20111), and informed written consent was provided by each participant at enrollment.

### 2.2. Sample Preparation and DNA Extraction

For genomic DNA extraction, the peripheral blood specimens from normal controls and CRC patients who enrolled in our study were collected. The EDTA containing tubes were used to preserve the samples of peripheral whole blood. The blood samples were centrifuged with the settings of 3000 rpm, 10 min, and the buffy coats from centrifuged whole blood specimens were extracted and further used for the DNA extraction [40,41]. The genomic DNA extraction assay was performed with QIAamp DNA blood mini kits following the protocols of the manufacturer’s manual to collect the DNA. The DNA elution was completed with the Tris-EDTA (TE) buffer, which was used to dissolve the DNA. Extracted DNA was further used as a DNA template in the real-time polymerase chain reactions (PCRs).

### 2.3. Selection of MMP-11 SNPs

In the current study, a total of five SNPs of *MMP-11* rs131451, rs738791, rs2267029, rs738792, and rs28382575 were selected based on the International HapMap Project database [33]. The *MMP-11* rs131451 SNP was selected because the *MMP-11* rs131451 polymorphisms were suggested to be associated with late-stage tumors and high-risk D’Amico classification in prostate cancer patients with biochemical recurrence [37]. The *MMP-11* rs738791 was selected because patients with the rs738791 polymorphic variant were observed to have greater risk of HCC compared with the wild-type (CC) carriers [35]. The MMP-11 SNP rs738792 was selected because the OSCC patients who carried at least one polymorphic C allele of *MMP-11* rs738792 showed an increased incidence of lymph node metastasis compared with those patients with homozygous T/T [34], and carriers who have at least one C allele of the *MMP-11* SNP rs738792 are likely to progress to Child–Pugh B or C grade in HCC patients [35]. The *MMP-11* SNP rs28382575 was selected because the HCC patients with at least one C allele of the *MMP-11* SNP rs28382575 are suggested to have a higher risk of developing stage III/IV disease, large tumors, or lymph node metastasis [35].

### 2.4. MMP-11 SNPs Genotyping

Assessment of allelic discrimination for the MMP-11 rs131451 (assay IDs: C___2213679_30), rs738791 (assay IDs: C___2448099_30), rs2267029 (assay IDs: C__15871447_20), rs738792 (assay IDs: C___2213764_20), and rs28382575 (assay IDs: C__61238655_10) SNPs was performed with an ABI StepOne Software v2.3 Real-Time PCR System. The TaqMan assay was used for the analysis of genotyping. The analysis and calculation of the final data of genotyping was processed with the SDS 7000 series software (Applied Biosystems, Foster City, CA, USA).

### 2.5. Statistical Analysis

To compare the age (years), gender, tumor location, tumor stage, tumor T status, lymph node status, metastasis, lymphovascular invasion, perineural invasion, and pathologic grading, the Chi-squared test or Student’s *t* test was performed between the patients with CRC and the controls. A statistical significance was suggested if *p* < 0.05. To compare the odds ratio (ORs) with their 95% confidence intervals (CIs) of the association between the genotypic frequencies and CRC risk, and the clinical pathological characteristics, the data was analyzed and assessed by the logistic regression models. All the data analysis in the current study was calculated and evaluated with SAS statistical software (Version 9.1, 2005; SAS Institute, Cary, NC, USA).

## 3. Results

### 3.1. Demographic and Clinical Characteristics of Study Cohorts

The distribution of demographical characteristics in 479 controls and 479 patients with CRC is demonstrated in Table 1. In our current study, we observed that the distribution of age (years) < 65 was 278 (58.0%) in controls and 251 (52.4%) in CRC patients, and the age ≧ 65 in controls and CRC patients was 201 (42.0%) and 228 (47.6%), respectively. For the distributions of gender, the male controls and CRC patients were 294 (61.4%) and 282 (58.9%), whereas the female controls and patients were 185 (38.6%) and 197 (41.1%), respectively. However, no statistically significant differences were found for age and gender between the CRC patients and the controls (Table 1).

### 3.2. MMP-11 Gene Polymorphisms were Associated with the Clinicopathological Characteristics of CRC

The genotype distributions of *MMP-11* gene polymorphisms in 479 controls and 479 patients with CRC are listed in Table 2. The highest distribution frequencies in patients with CRC of *MMP-11* genetic polymorphisms rs131451, rs738791, rs2267029, rs738792, and rs28382575 were heterozygous for TC, homozygous for CC, homozygous for GG, homozygous for TT, and homozygous for TT, respectively. Logistic regression models were adopted to estimate the odds ratios (ORs) and their 95% confidence intervals (CIs). After adjustment for the effects of age and gender, no significant associations were found between the CRC patients and the controls (Table 2).

**Figure 1:**
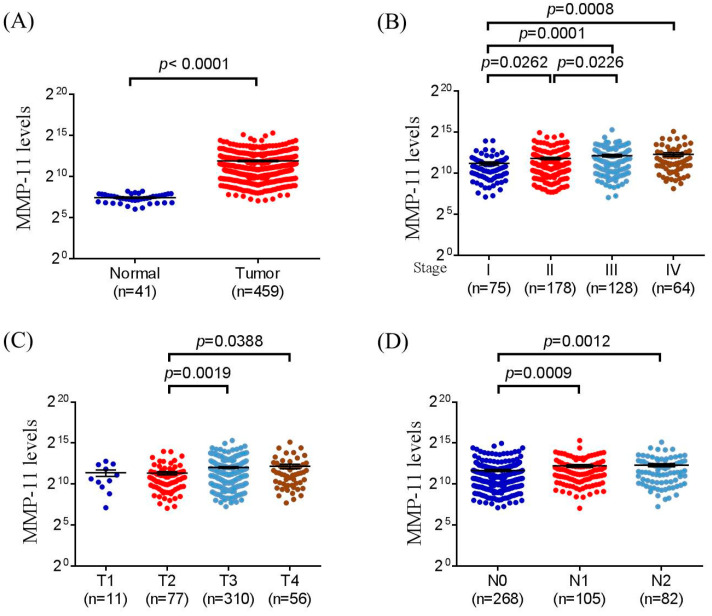
MMP-11 level of colorectal cancer patients from TCGA database. (**A**) MMP-11 levels were compared between the colorectal cancer tumor tissues and normal tissue. (**B**) MMP-11 levels were compared between stage I + II and stage III + IV. (**C**) MMP-11 levels were compared between the T1 + T2 stage and T3 + T4 stage. (**D**) MMP-11 levels were compared between the N0 stage and N1 stage.

**Figure 2:**
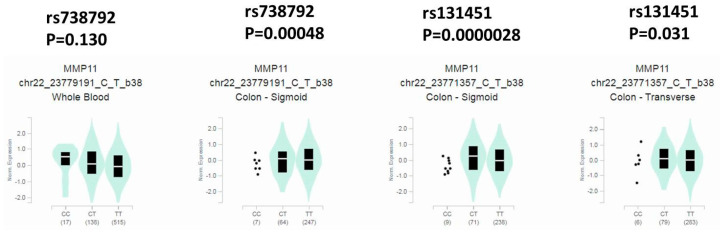
Distribution of MMP-11 expression in whole blood and colon (sigmoid, transverse) of *MMP-11* SNPs rs738792, rs131451 from Genotype-Tissue Expression (GTEx) database.

We further analyzed the distribution frequency of the clinical status and *MMP-11* genotype frequencies. In 369 of a total 479 CRC patients, a significant association was found in those individuals who carried the *MMP-11* rs738792 “TC + CC” genotypic variants, with a higher risk of perineural invasion of colon cancer patients after controlling for stages, tumor T status, lymph node status, metastasis, lymphovascular invasion, and cell differentiation (*p* = 0.025) (Table 3). We further analyzed the *MMP-11* polymorphisms of the clinical status in male and female CRC patients. The results demonstrated that the male CRC patients who carried the *MMP-11* rs131451 “TC + CC” genotype were associated with greater tumor T status (*p* = 0.028) and perineural invasion (*p* = 0.040) (Table 4). We further analyzed correlations of MMP-11 levels and their clinical parameters in CRC from The Cancer Genome Atlas (TCGA) dataset. We observed that MMP-11 expression was prone to be upregulated in colorectal carcinoma tissue compared with normal tissues (Figure 1A). Moreover, MMP-11 levels were also correlated with late stage, larger tumor sizes, and lymph node metastasis (Figure 1B–D). We further used the Genotype-Tissue Expression (GTEx) database to evaluate the correlations of *MMP-11* rs738792 and rs131451 SNPs to MMP-11 expression. The results of the GTEx database exhibited that individuals with the “C” allele of the *MMP-11* rs738792 (CC and CT) genotype were associated with higher MMP-11 expression in the sigmoid colon compared with the “TT” carriers (*p* = 0.00048) (Figure 1). For *MMP-11* rs131451 SNPs, individuals with the rs131451 polymorphisms “CC” and “CT” genotype were associated with higher MMP-11 expression in the sigmoid colon (*p* = 0.0000028) and transverse colon (*p* = 0.031), respectively, compared with the wild-type “TT” carriers (Figure 2).

The odds ratios (ORs) with their 95% confidence intervals were estimated by logistic regression models.

The adjusted odds ratios (AORs) with their 95% confidence intervals were estimated by multiple logistic regression models after controlling for age and gender.

## 4. Discussion

In this study, we demonstrated the associations between the *MMP-11* SNPs and CRC. The incidence of CRC is typically low at ages younger than 50 years but strongly increases with age [6,42]. Although the incidence of early-onset CRC (EOCRC) patients, referring to those individuals younger than 50 years, has been rapidly rising over the last 20 years [43,44,45], the median age at diagnosis of CRC is about 70 years in developed countries [6,46]. Previous study has suggested that the *MMP-11* polymorphisms and environmental carcinogens were associated with an increased risk for the development of OSCC [34]. Moreover, carriers of the CT + TT allele of the *MMP-11* rs738791 variant were suggested to possess greater risk of HCC compared with the wild-type (CC) carriers [35], and those with the *MMP-11* rs28382575 polymorphic “CT” genotype were found to be susceptible to UCC [38]. However, after we analyzed the genotype distributions of *MMP-11* polymorphisms in 479 controls and 479 patients with CRC, no statistically significant association was found between these two groups (Table 2), suggesting a limited effect of *MMP-11* polymorphisms for the susceptibility of CRC carcinogenesis.

We further analyzed the correlations between the *MMP-11* SNPs and clinical status of CRC, and we found that in 369 of a total 479 CRC patients, individuals who carried the *MMP-11* rs738792 “TC + CC” polymorphic variants were associated with higher risk of perineural invasion in colon cancer patients after controlling for clinical parameters (*p* = 0.025) (Table 3). Moreover, after we analyzed the *MMP-11* polymorphisms of the clinical status in CRC patients with different genders, we found that the male CRC patients who carried the *MMP-11* rs131451 “TC + CC” genotypic variants were correlated with greater tumor T status (*p* = 0.028) and perineural invasion (*p* = 0.040) (Table 4). Furthermore, numerous studies reported that perineurial invasion is associated with poor prognosis in CRC patients [47,48,49]. Therefore, the correlations among CRC prognosis and *MMP-11* polymorphism will be further investigated in our future work.

Generally, the colorectal cancer screening guidelines do not distinguish females from males, and sex specificity was not considered for interpretation in CRC despite the differences in tumor location between women and men [8,50,51]. Consistent with these results, the sex specificity was not significant between the CRC patients and controls in our study (*p* = 0.428) (Table 1). However, for the clinical status, results of the *MMP-11* rs131451 polymorphic variants showed discrepancy between male and female CRC patients (Table 4). Intriguingly, similar results of *MMP-11* rs131451 expression with sex differences were observed in our previous studies. The *MMP-11* SNPs, including the rs131451, have shown no impact on uterine cervical cancer in Taiwanese women [52], whereas the *MMP-11* rs131451 “TC + CC” polymorphic variants were correlated with advanced clinical stage (T stage; *p* = 0.007) and high-risk D’Amico classification (*p* = 0.015) in prostate cancer patients with biochemical recurrence [37]. Of note, analyses of TCGA revealed that MMP-11 levels were correlated with larger tumor sizes (Figure 1). Moreover, according to the data of the GTEx database, it was suggested that both the *MMP-11* rs131451 “CC + CT” genotypic variants expressed in the sigmoid colon or transverse colon were associated with higher MMP-11 expression compared with the wild-type “TT” carriers (Figure 2). However, in CRC, not only the male but also the female CRC patients with rs131451 “TC + CC” genotype were significantly associated with advanced tumor T status and perineural invasion (Table 4). In contrast, in the aspect of CRC prognostic biomarkers, it was suggested that there are clear sex differences in CRC characteristics, and sex-specific CRC prognostic biomarkers including ESM1, GUCA2A, and VWA2 for males and CLDN1 and FUT1 for female CRC patients were proposed [53]. One possible mechanism to explain this phenomenon was the interaction of sex hormones and their regulators in CRC. The sex hormones were suggested as contributors for gender disparity in incidence and mortality of CRC [54,55,56,57]. Notably, despite its ambiguous and contradictory role in CRC, testosterone was suggested to be involved in CRC development and prognosis [56,58], and the androgen was suggested to regulate MMPs (MMP-2) and the cellular processes of intimal hyperplasia [59]. Moreover, in the aspect of androgen receptor (AR), it was suggested that the expression of MMP-2 and MMP-9 was associated with the presence of AR in epithelial ovarian tumors [60], and the expressions of the MMP-11 and AR were significantly higher in cancer-associated fibroblasts (CAFs) from castration-resistant prostate tumors (CRPC) [61], suggesting a possible mechanism: that the higher MMP-11 expression resulting from *MMP-11* rs131451 polymorphisms might be associated with the induction of AR presence in CRC (Table 4) (Figure 1 and Figure 2). Men have a 20- to 25-fold higher testosterone production when compared with women [62,63], and the testosterone levels in women prior to menopause decline approximately 50% compared with their third decade [62,64]. Therefore, although the correlations and interactions of sex hormones with MMP-11 expression have remained unclear to date, it can be proposed that it is the AR presence induced by higher levels of MMP-11 expression which result from *MMP-11* rs131451 “CC + CT” polymorphisms, but also the direct interaction of testosterone and MMP-11, which is responsible for the discrepancy of sex specificity and poor prognosis in prostate cancer [61] and CRC [30,31] (Table 4) (Figure 2). In addition, for the application of MMPs as biomarkers in CRC detection, a previous study led by Koga et al. [65] demonstrated that the messenger RNA (mRNA) expression of *MMP-7* in the colonocytes isolated from feces was significantly higher in CRC patients than in healthy volunteers [65,66]. Moreover, numerous studies reported that expressions of MMP-14, MMP-17, and MMP-19 may be used as prognostic markers in CRC [67,68,69]. Therefore, a multi-SNP analysis for MMP-11, MMP-14, MMP-17, and MMP-19 will be investigated in our future work. Taken together, although the exact mechanisms and regulations remained incompletely understood, the *MMP-11* rs738792 and rs131451 SNPs may provide potential candidates for CRC biomarkers since these polymorphic variants were both linked with perineural invasion (Table 3 and Table 4) and higher expression of MMP-11 (Figure 1). Of note, the *MMP-11* rs131451 “TC + CC” genotype was further associated with greater tumor T status (*p* = 0.028) and perineural invasion (*p* = 0.040) in male CRC patients (Table 4), thereby providing a possible more detailed mechanism to explain the reason why the sex discrepancy of MMP-11 expression in CRC exists, and result in CRC disease development and prognosis with sex differences [31]. However, future well-designed studies are required to elucidate the exact mechanisms of *MMP-11* polymorphisms in CRC progression considering sex specificity, especially the detailed influences of sex hormones such as the decreasing levels of androgen and testosterone with age to MMP-11 expression in CRC disease progression and prognosis.

## 5. Conclusions

In conclusion, our results have demonstrated that despite the fact that *MMP-11* SNPs were not associated with CRC susceptibility, CRC patients who carried the *MMP-11* rs738792 “TC + CC” polymorphic variants were associated with higher risk of perineural invasion of the colon, and the male CRC patients who carried the *MMP-11* rs131451 “TC + CC” genotypic variants were associated with greater tumor T status and perineural invasion. The *MMP-11* rs738792 and rs131451 polymorphisms were also associated with higher MMP-11 expression either in the sigmoid colon or transverse colon. The *MMP-11* SNPs rs738792 and rs131451 may have potential to provide biomarkers to evaluate CRC disease progression, and the *MMP-11* rs131451 polymorphisms may shed light on sex discrepancy in CRC development and prognosis.

## Figures and Tables

**Table 1:** The distributions of demographical and clinical characteristics in 479 controls and 479 patients with CRC.

Variable	Controls (N = 479) n (%)	Patients (N = 479) n (%)	*p* Value
Age (yrs)			
<65	278 (58.0%)	251 (52.4%)	0.079
≥65	201 (42.0%)	228 (47.6%)	
Gender			
Male	294 (61.4%)	282 (58.9%)	0.428
Female	185 (38.6%)	197 (41.1%)	
Tumor location			
Rectum		110 (23.0%)	
Left colon		222 (46.3%)	
Right colon		147 (30.7%)	
Stage			
I + II		229 (47.8%)	
III + IV		250 (52.2%)	
Tumor T status			
T1–T2		116 (24.2%)	
T3–T4		363 (75.8%)	
Lymph node status			
N0		239 (49.9%)	
N1 + N2		240(50.1%)	
Metastasis			
M0		402 (83.9%)	
M1		77 (16.1%)	
Lymphovascular invasion			
No		267 (55.7%)	
Yes		212 (44.3%)	
Perineural invasion			
No		272 (56.8%)	
Yes		207 (43.2%)	
Pathologic grading			
Well		6 (1.3%)	
Moderately		437 (91.2%)	
Poorly		36 (7.5%)	

**Table 2:** Genotype distributions of *MMP-11* gene polymorphisms in 479 controls and 479 patients with CRC.

Variable	Controls (N = 479) n (%)	Patients (N = 479) n (%)	OR (95% CI)	AOR (95% CI)
rs131451				
TT	162 (33.8%)	161 (33.6%)	1.000 (reference)	1.000 (reference)
TC	234 (48.9%)	246 (51.4%)	1.058 (0.798–1.403)	1.065 (0.802–1.413)
CC	83 (17.3%)	72 (15.0%)	0.873 (0.595–1.281)	0.889 (0.605–1.306)
TC + CC	317 (66.2%)	318 (66.4%)	1.009 (0.772–1.319)	1.019 (0.779–1.333)
rs738791				
CC	234 (48.9%)	213 (44.5%)	1.000 (reference)	1.000 (reference)
CT	196 (40.9%)	214 (44.7%)	1.199 (0.917–1.569)	1.204 (0.920–1.575)
TT	49 (10.2%)	52 (10.9%)	1.166 (0.757–1.796)	1.202 (0.778–1.856)
CT + TT	245 (51.1%)	266 (55.5%)	1.193 (0.925–1.538)	1.203 (0.933–1.553)
rs2267029				
GG	266 (55.5%)	263 (54.9%)	1.000 (reference)	1.000 (reference)
GA	185 (38.6%)	188 (39.2%)	1.028 (0.789–1.340)	1.022 (0.784–1.333)
AA	28 (5.9%)	28 (5.9%)	1.011 (0.583–1.755)	1.033 (0.594–1.794)
GA + AA	213 (44.5%)	216 (45.1%)	1.026 (0.795–1.323)	1.024 (0.793–1.321)
rs738792				
TT	246 (51.4%)	241 (50.3%)	1.000 (reference)	1.000 (reference)
TC	195 (40.7%)	203 (42.4%)	1.063 (0.815–1.385)	1.070 (0.820–1.396)
CC	38 (7.9%)	35 (7.3%)	0.940 (0.575–1.538)	0.955 (0.583–1.564)
TC + CC	233 (48.6%)	238 (49.7%)	1.043 (0.809–1.343)	1.051 (0.815–1.355)
rs28382575				
TT	457 (95.4%)	446 (93.1%)	1.000 (reference)	1.000 (reference)
TC	22 (4.6%)	33 (6.9%)	1.537 (0.882–2.677)	1.596 (0.914–2.787)
CC	0 (0%)	0 (0.0%)	---	---
TC + CC	22 (4.6%)	33 (6.9%)	1.537 (0.882–2.677)	1.596 (0.914–2.787)

**Table 3:** Distribution frequency of the clinical status and *MMP-11* rs738792 genotype frequencies in 479 CRC patients.

Variable	All (N = 479)			Rectum (N = 110)			Colon (N = 369)		
	TT (N = 241)	TC + CC (N = 238)	*p* Value	TT (N = 60)	TC + CC (N = 50)	*p* Value	TT (N = 181)	TC + CC (N = 188)	*p* Value
Stages									
I + II	115 (47.7%)	114 (47.9%)	*p* = 0.892	31 (51.7%)	29 (58.0%)	*p* = 0.482	84 (46.4%)	85 (45.2%)	*p* = 0.945
III + IV	126 (52.3%)	124 (52.1%)		29 (48.3%)	21 (42.0%)		97 (53.6%)	103 (54.8%)	
Tumor T status									
T1 + T2	62 (25.7%)	54 (22.7%)	*p* = 0.805	20 (33.3%)	16 (32.0%)	*p* = 0.569	42 (23.2%)	38 (20.2%)	*p* = 0.885
T3 + T4	179 (74.3%)	184 (77.3%)		40 (66.7%)	34 (68.0%)		139 (76.8%)	150 (79.8%)	
Lymph node status									
Negative	119 (49.4%)	120 (50.4%)	*p* = 0.630	32 (53.3%)	30 (60.0%)	*p* = 0.411	87 (48.1%)	90 (47.9%)	*p* = 0.643
Positive	122 (50.6%)	118 (49.6%)		28 (46.7%)	20 (40.0%)		94 (51.9%)	98 (52.1%)	
Metastasis									
Negative	204 (84.6%)	198 (83.2%)	*p* = 0.955	46 (76.7%)	45 (90.0%)	*p* = 0.090	158 (87.3%)	153 (81.4%)	*p* = 0.212
Positive	37 (15.4%)	40 (16.8%)		14 (23.3%)	5 (10.0%)		23 (12.7%)	35 (18.6%)	
Lymphovascular invasion									
No	136 (56.4%)	131 (55.0%)	*p* = 0.457	38 (63.3%)	33 (66.0%)	*p* = 0.830	98 (54.1%)	98 (52.1%)	*p* = 0.426
Yes	105 (43.6%)	107 (45.0%)		22 (36.7%)	17 (34.0%)		83 (45.9%)	90 (47.9%)	
Perineural invasion									
No	147 (61.0%)	125 (52.5%)	*p* = 0.051	39 (65.0%)	34 (68.0%)	*p* = 0.998	108 (59.7%)	91 (48.4%)	*p* = 0.025 ^a^
Yes	94 (39.0%)	113 (47.5%)		21 (35.0%)	16 (32.0%)		73 (40.3%)	97 (51.6%)	
Cell differentiation									
Well/Moderately	227 (94.2%)	216 (90.8%)	*p* = 0.164	60 (100%)	49 (98.0%)	-----	167 (92.3%)	167 (88.8%)	*p* = 0.323
Poorly	14 (5.8%)	22 (9.2%)		0 (0.0%)	1 (2.0%)		14 (7.7%)	21 (11.2%)	

^a^ AOR (95% CI):1.783 (1.074–2.960). The adjusted odds ratios (AORs) with their 95% confidence intervals were estimated by multiple logistic regression models after controlling for stages, tumor T status, lymph node status, metastasis, lymphovascular invasion, perineural invasion, and cell differentiation.

**Table 4:** Distribution frequency of the clinical status and *MMP-11* rs131451 genotype frequencies in 479 CRC patients with different genders.

Variable	All (N = 479)			Male (N = 282)			Female (N = 197)		
	TT (N = 161)	TC + CC (N = 318)	*p* Value	TT (N = 96)	TC + CC (N = 186)	*p* value	TT (N = 65)	TC + CC (N = 132)	*p* Value
Stages									
I + II	80 (49.7%)	149 (46.9%)	*p* = 0.317	51 (53.1%)	94 (50.5%)	*p* = 0.812	29 (44.6%)	55 (41.7%)	*p* = 0.134
III + IV	81 (50.3%)	169 (53.1%)		45 (46.9%)	92 (49.5%)		36 (55.4%)	77 (58.3%)	
Tumor T status									
T1 + T2	47 (29.2%)	69 (21.7%)	*p* = 0.216	34 (35.4%)	43 (23.1%)	*p* = 0.028 ^a^	13 (20.0%)	26 (19.7%)	*p* = 0.999
T3 + T4	114 (70.8%)	249 (78.3%)		62 (64.6%)	143 (76.9%)		52 (80.0%)	106 (80.3%)	
Lymph node status									
Negative	81 (50.3%)	158 (49.7%)	*p* = 0.238	52 (54.2%)	99 (53.2%)	*p* = 0.545	29 (44.6%)	59 (44.7%)	*p* = 0.172
Positive	80 (49.7%)	160 (50.3%)		44 (45.8%)	87 (46.8%)		36 (55.4%)	73 (55.3%)	
Metastasis									
Negative	135 (83.9%)	267 (84.0%)	*p* = 0.380	84 (87.5%)	157 (84.4%)	*p* = 0.663	51 (78.5%)	110 (83.3%)	*p* = 0.152
Positive	26 (16.1%)	51 (16.0%)		12 (12.5%)	29 (15.6%)		14 (21.5%)	22 (16.7%)	
Lymphovascular invasion									
No	95 (59.0%)	172 (54.1%)	*p* = 0.942	59 (61.5%)	104 (55.9%)	*p* = 0.697	36 (55.4%)	68 (51.5%)	*p* = 0.554
Yes	66 (41.0%)	146 (45.9%)		37 (38.5%)	82 (44.1%)		29 (44.6%)	64 (48.5%)	
Perineural invasion									
No	99 (61.5%)	173 (54.4%)	*p* = 0.341	66 (68.8%)	104 (55.9%)	*p* = 0.040 ^b^	33 (50.8%)	69 (52.3%)	*p* = 0.849
Yes	62 (38.5%)	145 (45.6%)		30 (31.2%)	82 (44.1%)		32 (49.2%)	63 (47.7%)	
Cell differentiation									
Well/Moderately	154 (95.7%)	289 (90.9%)	*p* = 0.096	91 (94.8%)	165 (88.7%)	*p* = 0.129	63 (96.9%)	124 (93.9%)	*p* = 0.371
Poorly	7 (4.3%)	29 (9.1%)		5 (5.2%)	21 (11.3%)		2 (3.1%)	8 (6.1%)	

^a^ AOR (95% CI):1.254 (1.025–1.534); ^b^ AOR (95% CI):1.773 (1.027–3.062). The adjusted odds ratios (AORs) with their 95% confidence intervals were estimated by multiple logistic regression models after controlling for stages, tumor T status, lymph node status, metastasis, lymphovascular invasion, perineural invasion, and cell differentiation.

## Data Availability

The data presented in this study are available on request from the corresponding author.
